# A Novel Approach for Powder Bed Fusion of Ceramics Using Two Laser Systems

**DOI:** 10.3390/ma16062507

**Published:** 2023-03-21

**Authors:** Duran Kaya, Mohamed Abdelmoula, Gökhan Küçüktürk, David Grossin, Artemis Stamboulis

**Affiliations:** 1 Department of Mechanical Engineering, Graduate School of Natural and Applied Sciences, Gazi University, Ankara 06570, Turkey; 2 School of Engineering and Applied Science, University of Wisconsin-Milwaukee, Milwaukee, WI 53211, USA; 3 Department of Mechanical Engineering, Gazi University, Ankara 06500, Turkey; 4 Centre Inter-Universitaire de Recherche et d’Ingénierie des Matériaux, Université de Toulouse, CNRS, INP- ENSIACET, 4 Allée Émile Monso, CEDEX 4, 31432 Toulouse, France; 5 School of Metallurgy and Materials, University of Birmingham, Birmingham B15 2TT, UK

**Keywords:** PBF, preheating, alumina, simulation, process parameters

## Abstract

The one-step AM process is considered the goal many researchers seek in the field of Additive Manufacturing (AM) of high-technology ceramics. Among the several AM techniques, only Powder Bed Fusion (PBF) can directly print high-technology ceramics using one step. However, the PBF technique faces numerous challenges to efficiently be employed in the PBF of ceramics. These challenges include the formation of cracks, generated thermal stress, effective laser–powder interaction, and low acquired relative density. This study developed a new preheating mechanism for ceramic materials using two laser systems to surpass beyond these challenges and successfully print ceramics with a single-step AM method. One laser is used to preheat the powder particles before the second laser is utilised to complete the melting/sintering process. Both lasers travel along the same scanning path. There is a slight delay (0.0001 s) between the preheating laser and the melting/sintering laser to guarantee that the melting/sintering laser scans a properly preheated powder. To further facilitate testing of the preheating system, a numerical model has been developed to simulate the preheating and melting process and to acquire proper process parameters. The developed numerical model was shown to determine the correct process parameters without needing costly and time-consuming experiments. Alumina samples (10 × 10 × 6 mm^3^) were successfully printed using alumina powder as feedstock. The surface of the samples was nearly defect-free. The samples’ relative densities exceeded 80%, the highest reported relative density for alumina produced by a single-step AM method. This discovery can significantly accelerate the transition to a one-step AM process of ceramics.

## 1. Introduction

Ceramics are an important class of materials widely used in aerospace and medical sectors due to their superior mechanical and physical properties [1]. These properties include high compressive strength, high elastic modulus, low thermal conductivity, low density, high hardness, and friction resistance [2,3,4,5]. Ceramic materials can be manufactured using conventional methods such as slip casting, extrusion, injection moulding, and pressing [6,7,8,9]. However, conventional techniques cannot cope with the current manufacturing revolution that involves the need to manufacture highly complex designs. To solve this problem, Additive Manufacturing (AM) can be used due to its ability to manufacture highly complex designs [10,11,12]. AM is a trending manufacturing technology that can produce parts from 3D CAD models layer by layer [13]. The AM process includes seven techniques defined by the ISO/ASTM 52900 [13]: Powder Bed Fusion (PBF), binder jetting, vat-photopolymerisation, extrusion, direct energy deposition, material jetting, and sheet lamination.

Currently, AM of ceramics is witnessing rapid development either in the feedstock or the application of AM techniques. Many studies focused on the AM of ceramic materials using specific techniques such as binder jetting [14,15,16,17,18,19,20], extrusion [21,22,23,24,25], and PBF. For example, Gonzalez et al. [26] used a binder jetting technique to manufacture dense alumina parts investigating different build parameters and sintering techniques. They reached a 96% relative density, but the main issue was the shrinkage in the part’s dimensions (almost a 10% reduction of the part dimension). Additionally, recently Chen et al. [27] also investigated the binder jetting of alumina. Different factors were studied, including the powder properties, powder bed properties, and process parameters. The measured relative density was 64.2%, and the shrinkage was about 15%. On the other hand, Yu et al. [28] manufactured yttria partially stabilised zirconia by extrusion, and they obtained zirconia parts with good mechanical properties. However, the shrinkage in the part dimensions was still high. He et al. [29] reported an extrusion system for the AM of zirconia, and they achieved a relative density of 99%, but the shrinkage of 20% was still high.

To conclude, the application of binder jetting in AM of ceramics needs post-processing operations to remove the binder and sinter the powder particles together. These post-processes lead to shrinkage in the part’s dimensions. In the case of extrusion, powder preparation to form the slurry is needed before printing and post-processing operations are also required to sinter the powder and obtain the final part. Therefore, the initial powder preparations and the post-processing operations are considered costly.

In order to push the development of the AM of ceramics, the initial feedstock preparations and post-processing operations should be removed from the manufacturing cycle (using a one-step AM process). This can be achieved using the PBF technique. PBF can print ceramic materials directly using pure ceramic powder and without post-processing to reach the final shape [30]. However, some problems hinder the application of PBF to print ceramic materials, such as high melting/sintering points, thermal shocks, and developed thermal stresses and cracks [31]. Many previous studies [32,33,34,35] suggested preheating the powder layer before scanning with another laser system to overcome these problems. Yves-Christian et al. [36] preheated the powder layer using a CO2 laser before scanning with an Nd:YAG laser to control the developed thermal stress and cracks. They succeeded in reducing the developed cracks by increasing the preheating temperature. By developing a preheating system, Liu et al. [37] preheated yttria-stabilised zirconia (YSZ) powder’s top surface. The system consists of an Nd:YAG laser to preheat the powder and another fibre laser for scanning. As a result, they could print YSZ parts with a relative density of 84% with fewer cracks. Reviewing the literature regarding the preheating systems used during PBF of ceramic materials, it was noticed that previous studies focused on preheating a large area of the powder layer or preheating the baseplate [36,37]. Preheating a large area of the powder bed cannot ensure that all scanned particles will be preheated to the same temperature, and it is also considered an energy-wasting method.

In this study, we developed a novel preheating system consisting of two laser sources, one for preheating and the other for melting/sintering particles, and the entire idea is described in detail in the following sections.

The study started with developing a numerical model for the two lasers to determine the proper process parameters for each laser source (laser power, scanning speed, and hatching distance) at the beginning. This can save time and effort and directly obtain the proper process parameters instead of using hit and trial, which is time- and cost-consuming. Next, alumina powder was used as the feedstock to test the developed system, and alumina cubic samples (10×10×6 mm^3^) were built using the developed experimental setup. Finally, the samples were evaluated and characterised.

## 2. Materials and Methods

**Figure 1:**
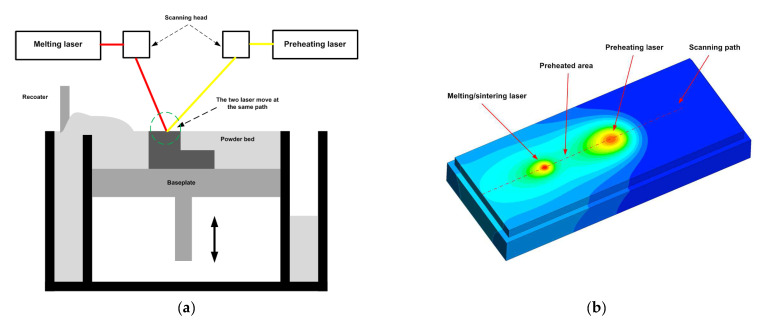
The preheating system developed in this study; (**a**) schematic diagram for the system, (**b**) simulation of the two lasers moving at the same scanning path.

This study developed a new preheating system for the PBF of ceramic materials to control and eliminate the development of thermal stresses and cracks. A CO_2_ laser is used for preheating, while a fibre laser is used for melting/sintering the powder particles. The two lasers move together on the powder layer, following the scanning path shown in Figure 1. The CO_2_ laser precedes the fibre laser by 0.0001 s to preheat the powder particles. By using this system, the preheating of the powder is ensured. Additionally, this preheating system can control and reduce the temperature gradient and, as a result, reduce the generated thermal stress and cracks.

### 2.1. Numerical Method

The melting/sintering of powder particles is a complex process, and it is challenging to model mathematically. Therefore, some assumptions should be made to simulate the melting/sintering process of the powder. These assumptions are as follows: (1) the heat distribution of the laser beam is uniform, (2) the top surface of the molten pool is flat, (3) no heat losses occur by evaporation, and (4) the powder bed is continuous and homogeneous. All heat transfer mechanisms (conduction, radiation, and convection) are considered during the model development. Two mechanisms occur when the laser scans a powder bed with a specific power and speed; the powder particles absorb a portion of the laser beam while the other portion is back-scattered into the surroundings.

#### 2.1.1. Numerical Model Development

There are two laser systems: one for preheating and the other for melting/sintering the powder particles. Heat is transferred to the powder particles when the laser scans them. To simulate heat transfer, the energy equation below (Equation (1)) is used to describe the heat transfer according to Moser et al. [38]:

(1) $$\rho C_{p}\frac{\partial T}{\partial t}=\nabla .\left(k\;\nabla \;T\right)+S_{H}+S_{M/S}$$

where $\rho ,\;C_{p},T$, k, and t are the density, specific heat of alumina, temperature, the thermal conductivity of powder, and time of the process, respectively. The terms $S_{H}$ and $S_{M/S}\;$in Equation (1) refer to the laser heat source per unit volume (W/m^3^) for preheating and melting/sintering, respectively. According to [38], the preheating laser source $S_{H}$ and melting/sintering source $S_{M/S}$ can be described as follows:

(2) $$S_{H}=A_{H}I_{H}\alpha_{H}\;\exp\left(-2\;\frac{{\left(x-v_{x}t\right)}^{2}+{\left(y-v_{y}t\right)}^{2}}{R^{2}}-\alpha_{H}z\right)$$

(3) $$S_{M/S}=A_{M/S}I_{M/S}\alpha_{M/S}\;\exp\left(-2\;\frac{{\left(x-v_{x}t\right)}^{2}+{\left(y-v_{y}t\right)}^{2}}{R^{2}}-\alpha_{M/S}z\right)$$

(4) $$I_{o}=\frac{p_{H}}{\pi \;R_{H}{}^{2}}$$

(5) $$I_{o}=\frac{p_{M/S}}{\pi \;R_{M/S}{}^{2}}$$

where *A*, *I*, α, v, and *R* are the material absorptivity, laser intensity, absorption coefficient, scanning speed, and laser radius, respectively. The absorption coefficient was estimated according to [39,40]. The x and y parameters in Equations (2) and (3) were used to control the laser movement on the powder bed. A User-Defined Function (UDF) was developed to model these two heat sources in the energy equation. ANSYS FLUENT 2020R2 was used to solve the developed numerical model.

**Figure 2:**
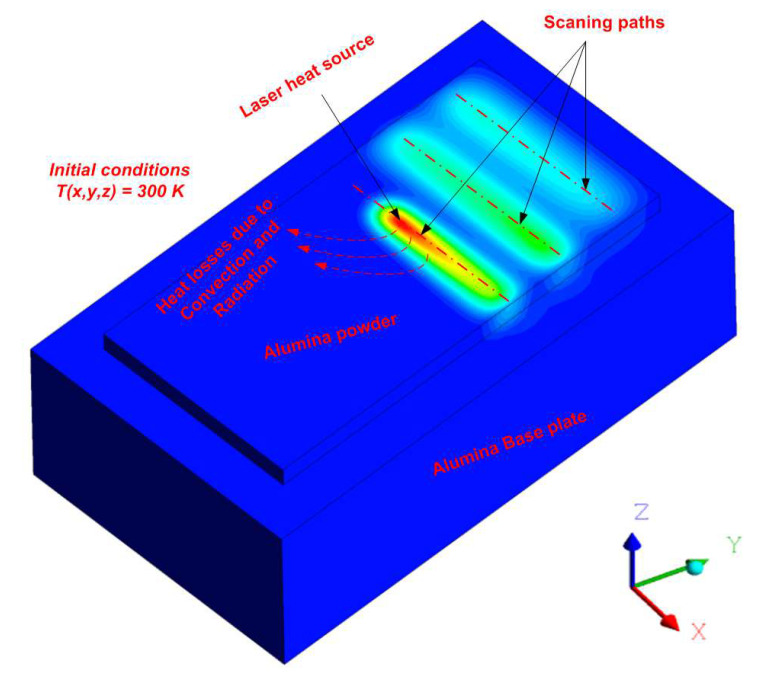
The initial and boundary conditions used in this study.

It is worth mentioning that the initial and boundary conditions were considered in this study, as shown in Figure 2 and described by Equations (6) and (7):

(6) $$T{\left(x,y,z\right)}_{t=0}=T_{O}$$

(7) $$-k(\frac{\partial T}{\partial z})=\dot{S_{h}}-h_{cov}\left(T_{a}-T_{s}\right)-\sigma \varepsilon \left(T_{a}^{4}-T_{s}^{4}\right)$$

where $T_{a}$ is the ambient temperature in °C, $T_{s}$ is the powder surface temperature in °C, *σ* is the Stefan–Boltzmann constant, ℇ is the emissivity, and $h_{cov}$ is the convection coefficient.

To consider the effect of temperature on the physical properties of alumina during laser preheating and scanning, material properties as a function of temperature (as described in Table 1) and other constants, according to Fan et al. [41], were considered in the developed UDF.

Additionally, the phase transformation from solid to liquid and liquid to solid was considered in the developed UDF. This was achieved by using the enthalpy technique described by ANSYS Inc [43]. The material enthalpy H is defined as the sum of the internal energy of the system U and the product of the pressure P multiplied by the volume V as described by Equation (8), or the amount of sensible heat h and latent heat content in the system ΔH as described by Equation (9).

H = U + P·V(8)

H = h + ΔH(9)

where U is the internal energy, P, V, h, and ΔH are the internal energy, pressure, change in volume, sensible heat, and latent heat, respectively. The sensible heat (h) and the latent heat (ΔH) can be described as follows:

(10) $$h\;=\;\mathrm{href}\;+\int_{T_{ref}}^{T}C_{p}\;\Delta T$$

ΔH = β L(11)

where h_ref_ is the reference enthalpy, $\;C_{p}$ is the specific heat, and β is the liquid fraction in the material. The liquid fraction (β) can be estimated by the equation below [43]:

(12) $$\beta =\frac{T-T_{solidus}}{T_{liquidus}-T_{solidus}}$$

The energy equation, Equation (1), can be solved for the temperature distribution through the solution domain and then be used to measure β, which defines the melting and solidification phase within the solution domain according to Equation (13) below. Additionally, it is worth mentioning that a User-Defined Memory (UDM) available in ANSYS FLUENT was used to monitor the molten region during the laser scanning of the powder.

(13) $$\beta =\{\begin{array}{c} <1\\ =0\\ >1 \end{array}\begin{array}{c} solid\;region\\ transition\;region\\ melting\;region \end{array}$$

#### 2.1.2. Numerical Solution

**Figure 3:**
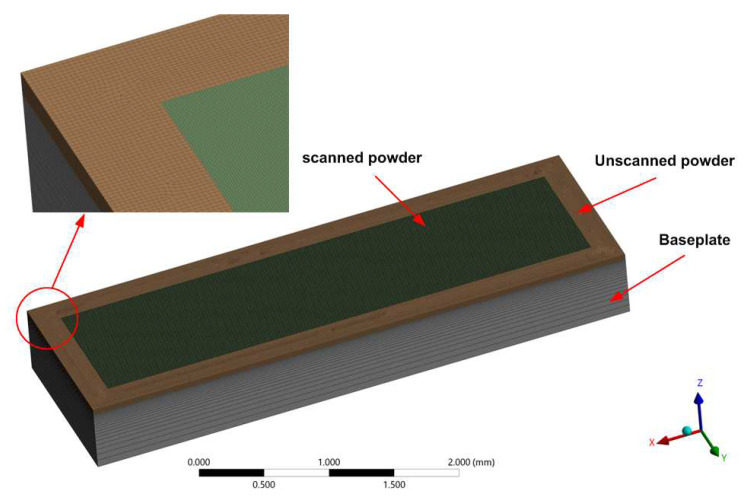
The model geometry and the computational domain (the mesh) were used in the study.

The model geometry and the computational domain used in this study are shown in Figure 3, and its dimensions are summarised in Table 2. ANSYS Design Modeller was used to create the model geometry. The model geometry consists of the powder layer, the surrounding powder, and the baseplate. Only one layer was considered for the analysis as the same behaviour was repeated at each successive layer. ANSYS Mesher was used to create the computational domain (the mesh). The mesh size was selected to be very fine in the powder region and coarse in the surrounding powder and the baseplate regions, as shown in Figure 3.

**Figure 4:**
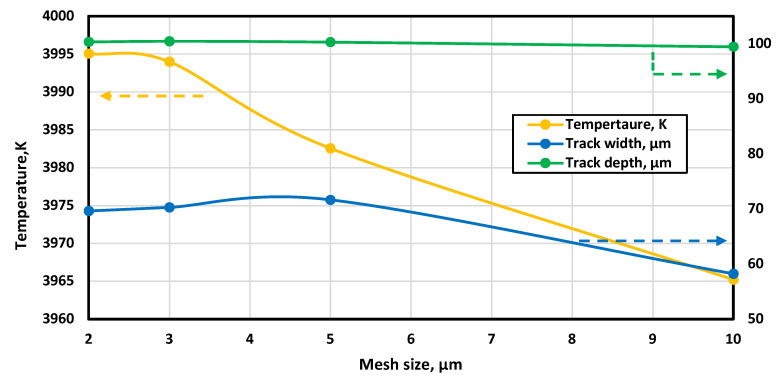
Mesh density analysis result.

**Figure 5:**
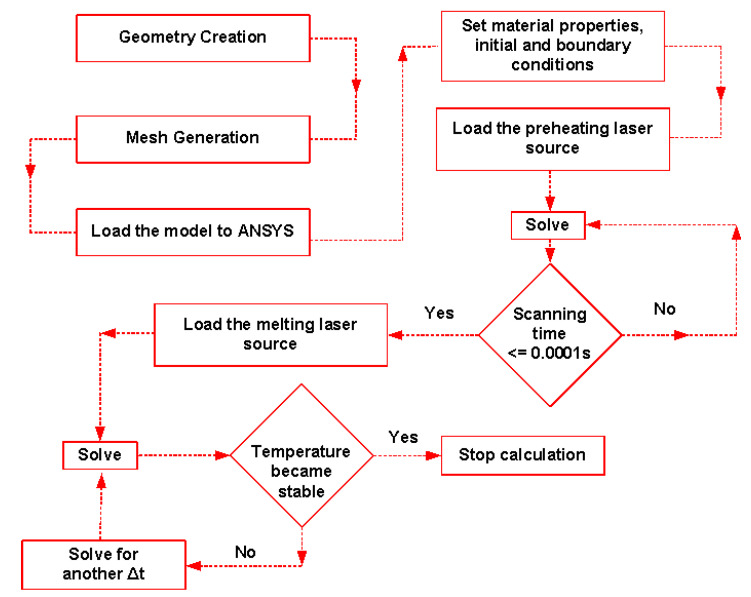
Flowchart of the numerical model solution procedure.

A mesh density analysis was carried out to avoid any error from bad-quality meshing. Four different mesh sizes, 10, 5, 3, and 2 µm, were checked. Three criteria were used to evaluate the mesh quality: the maximum temperature, the melting track depth, and its width. The results are summarised in Figure 4. The maximum temperature, and track depth and width were stable with any mesh size after 3 µm. Therefore, a mesh sizing of 2 µm was used as the mesh sizing. Figure 5 shows the numerical solution procedure followed during the solution of the numerical model.

### 2.2. Experimental Method

#### 2.2.1. Feedstock Material

**Figure 6:**
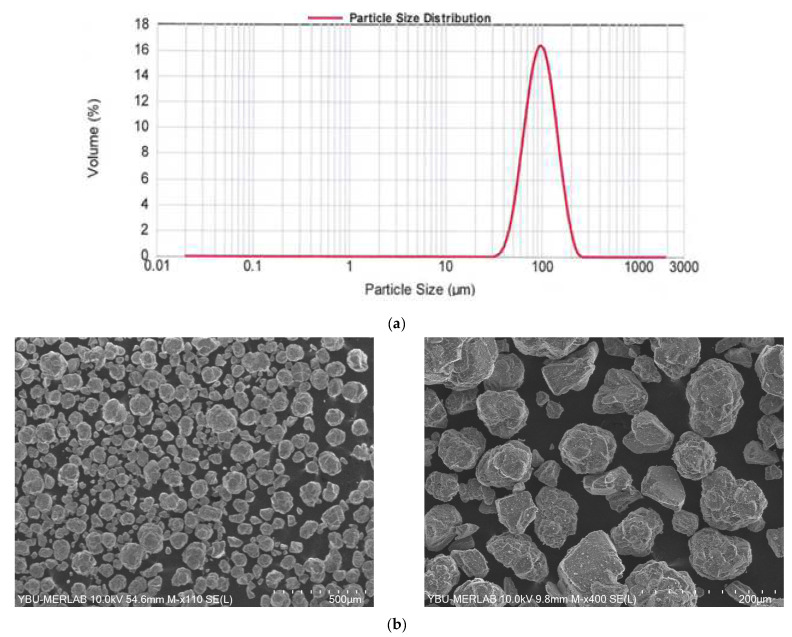
Alumina powder: (**a**) particle size distribution, (**b**) SEM images.

The feedstock used in this study is alumina powder (α-alumina 95% purity) supplied from Nanografi Nanotechnology (Turkey) with d_50_ = 95 µm. The particle size distribution of the powder is shown in Figure 6a. Figure 6b shows the SEM images for the powder particles with an irregular shape, which can induce problems during layer deposition as the irregular powder shapes can increase inter-particle friction. Trails were made to check the layer deposition, and it was found that the powder was deposited without any defect or problem.

#### 2.2.2. The Developed Experimental Setup

**Figure 7:**
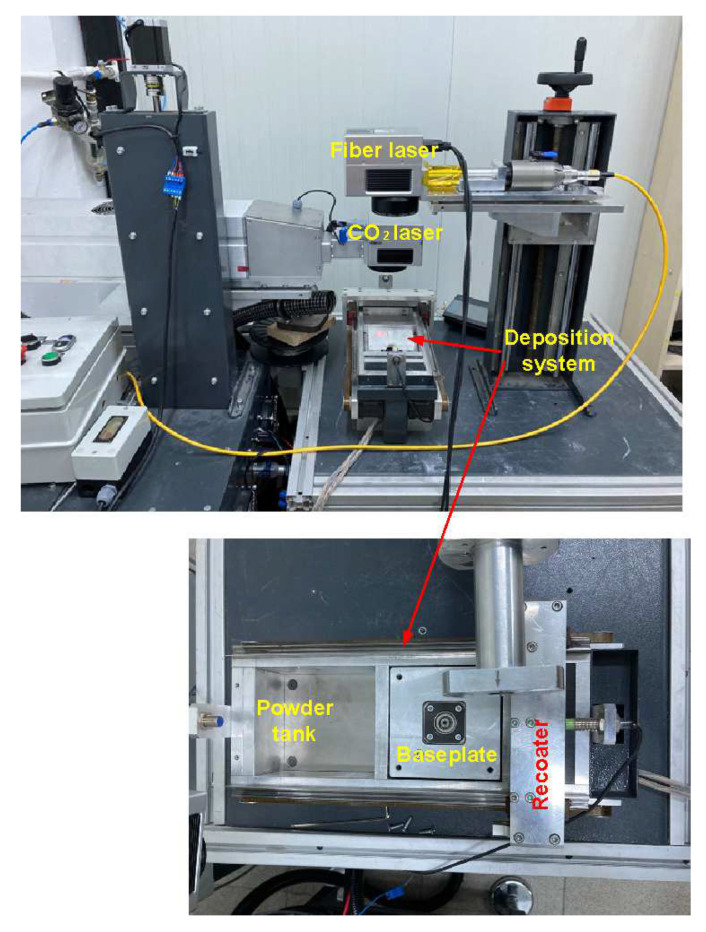
The developed experimental setup.

The experimental setup developed in this study is shown in Figure 7. There are two laser systems: a CO_2_ laser for preheating and a fibre laser for melting/sintering the powder particles. The specifications of the laser sources are summarised in Table 3. A simple system was also developed for layer deposition where there is a tank for the powder feed and another tank for the excess powder. The baseplate is located between the two tanks, as shown in Figure 7.

#### 2.2.3. Sample Preparation and Characterisation Methods

**Figure 8:**
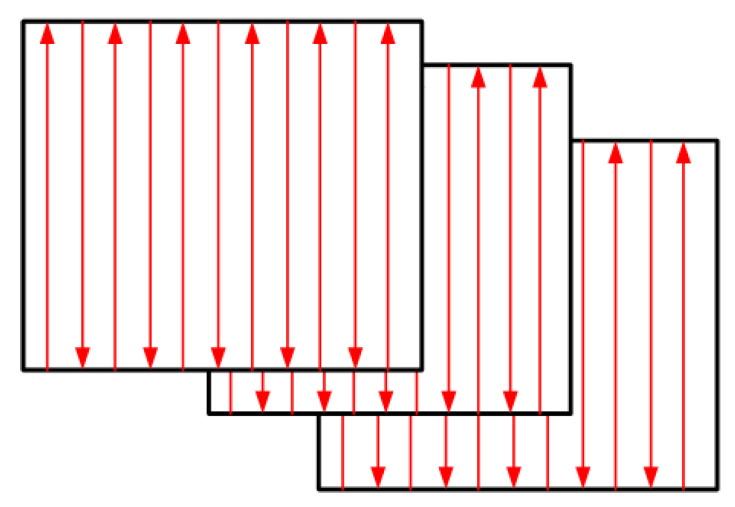
A zig-zag scanning strategy was used in this study as recommended by Abdelmoula et al. [44].

Alumina samples (10 × 10 × 6 mm^3^) were printed using the developed experimental setup. The developed numerical model was used to determine the process parameters (laser power, scanning speed, and hatching distance) used for sample printing using the zig-zag scanning strategy (as recommended by Abdelmoula et al. [44]) shown in Figure 8. Characterisation techniques were used to characterise the printed samples, such as relative density measurements, SEM imaging, X-ray Diffraction (XRD), and EDS analysis. The density was measured using Archimedes’ method. The HITACHI SU500 was used for the SEM and EDS study. The BRUKER D8 Advance (D8-Advance, BRUKER, US) diffractometer was used for the XRD analysis.

## 3. Results

### 3.1. Numerical Model Validation

**Figure 9:**
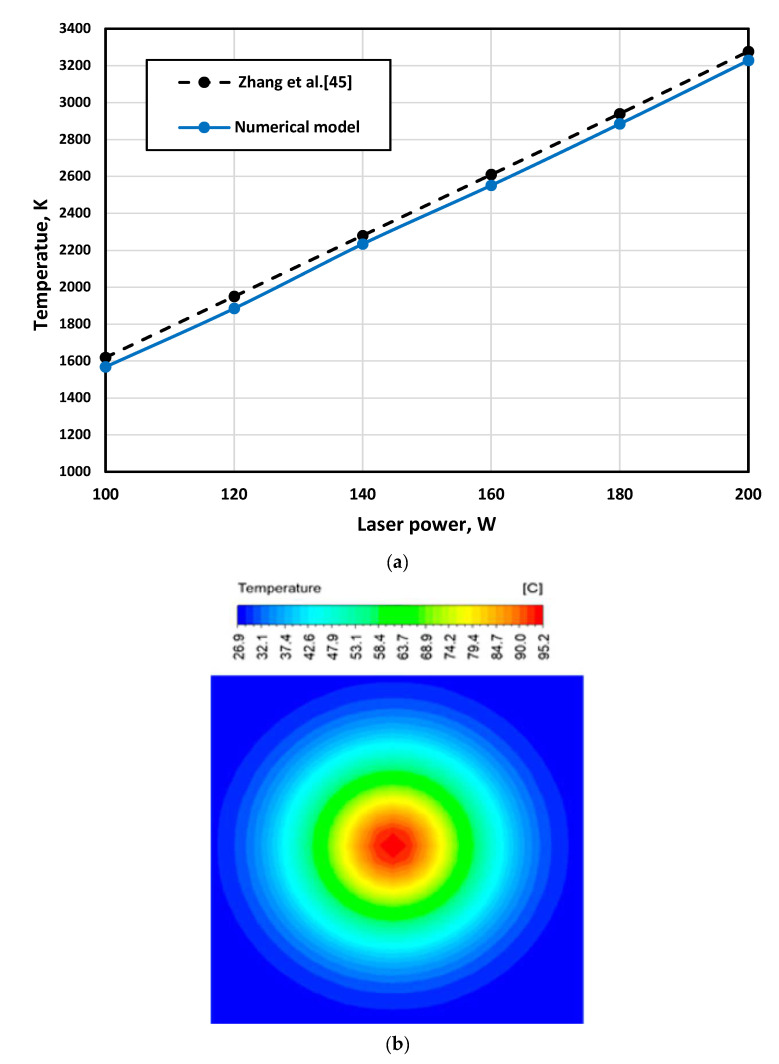
Validation results of the developed model: (**a**) comparison of the maximum temperature obtained from the numerical model by Zhang et al. [45] and mesh density analysis, (**b**) comparison of the temperature contour obtained from the numerical model with the reported contour by Moser et al. and Edith Wiri et al. [38,46].

It was challenging to carry out enough trials to achieve a successful building to validate the developed numerical model; instead, the numerical model has been validated with available experimental data to effectively use it in selecting the appropriate parameters. Therefore, the maximum obtained temperature to form the numerical model was compared with the available data from Zhang et al. [45], as shown in Figure 9a. Zhang et al. [45] validated their model initially by comparing the temperature history obtained from the model with an experimental temperature history obtained through an infrared thermal imager. The comparison between the developed model and the model of Zhang et al. [45] showed a good agreement between the two results. However, the maximum error between them was 3.34%. Then, another validation was carried out to confirm the model validation. The temperature contour, as shown in Figure 9b, was obtained by the numerical model using conditions close to those used in the studies reported by Moser et al. [38] and Edith Wiria et al. [46]. The contour captured using a thermal camera [38,46] confirmed a good agreement with a calculation error of 1.24%.

### 3.2. Numerical Results

#### 3.2.1. Laser Power and Scanning Speed

The proper values of the process parameters (laser power, scanning speed, hatching distance, and layer thickness) are essential to successful printing. The laser power and scanning speed define the amount of heat transferred to the powder particles. The laser power indicates the heat transferred per second, while the scanning speed controls the rate by which the heat is distributed over the powder layer. High laser power with low scanning speed leads to pores inside the printed part, while low laser power with increased scanning speed leads to insufficient melting/sintering of powder particles. Therefore, it is essential to maintain an equilibrium between the laser power and scanning speed [47]. Thus, the numerical model was used to determine the proper value of the laser power and scanning speed by considering 60 µm as a layer thickness (the layer thickness was selected based on the deposition system as it was challenging to use a layer thickness value below 60 µm). The scanning speed was fixed at 150 mm/s, and the power of the preheating and melting lasers was calculated accordingly to test the experimental setup and investigate how effective it was for the PBF of ceramic materials.

**Figure 10:**
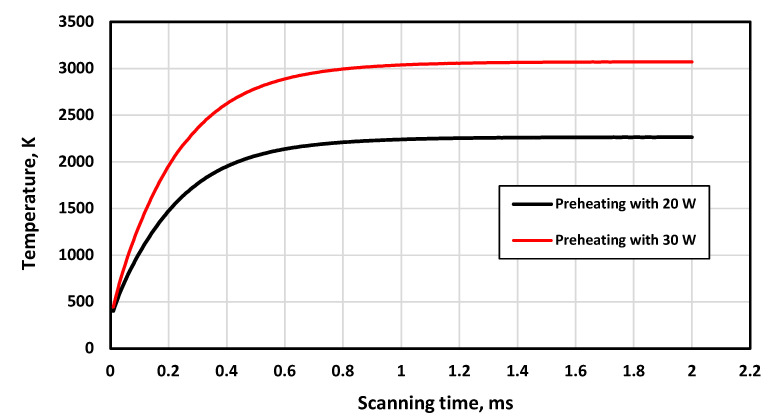
Temperature history for the preheating laser source using different power.

For the preheating laser source, the laser power was calculated based on achieving a preheating temperature range of 2000 K to 3000 K. This temperature was selected based on the results described by Liu et al. [37]. They found that increasing the preheating temperature significantly reduced the developed cracks in the printed part. The developed model was used to calculate the preheating laser power range, and it was found that the laser power of 20 W to 30 W satisfied the previous condition, as shown in Figure 10.

After selecting the proper power range for the preheating laser, the developed model was used to calculate the proper melting laser power based on adhering the current layer to the layer below. It was found that using a melting laser power of 150 W satisfied the conditions mentioned above, and the results are summarised in Table 4. In addition, the maximum temperature during scanning did not exceed the alumina evaporation point.

#### 3.2.2. Hatching Distance

**Figure 11:**
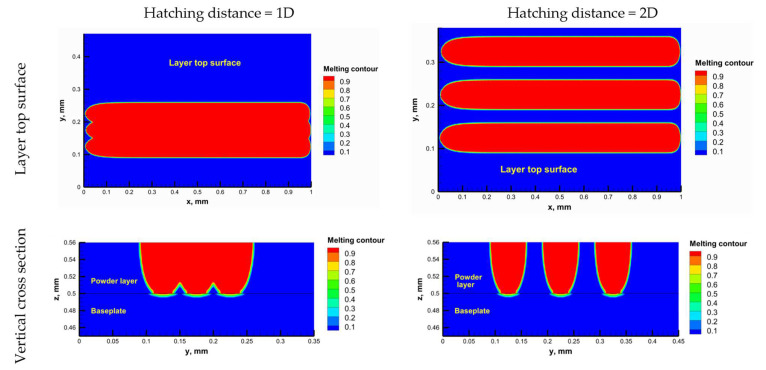
The top surface melting contour for different hatching distances.

The hatching distance refers to the distance between two adjacent scanning traces. It is crucial to ensure overlap between the adjacent traces to obtain good mechanical properties and avoid insufficient melting between adjacent traces [47]. The numerical model was used to test different hatching distances and select an appropriate value. Figure 11 shows the melting contour for adjacent traces using hatching distances of 1 × D and 2 × D (where D is the melting laser spot size) at the layer top surface and a vertical cross-section through the layer. Using the hatching distance 2 × D, the adjacent traces were not connected, and there was insufficient melting between them. This can adversely affect the mechanical properties of the printed part. On the other hand, the adjacent traces were almost entirely connected using a hatching distance of 1 × D, and sufficient melting occurred between them. Additionally, previous studies [44,48] reported that in PBF of ceramics, the hatching distance should be 0.5 × D for successful printing, but the preheating system developed in this study enabled the hatching distance to be increased up to 1 × D which will decrease the building time by half. This is considered an important advantage for the developed preheating system.

### 3.3. Experimental Results

#### 3.3.1. PBF of Alumina Samples

**Figure 12:**
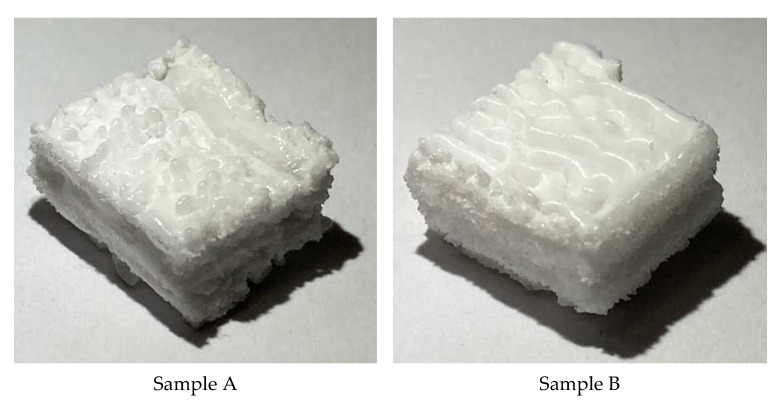
Alumina samples (10 × 10 × 6 mm^3^) printed using the parameters summarised in Table 5 with 20 W preheating laser power (Sample A) and 30 W (Sample B),

After obtaining the proper process parameters from the numerical model, two alumina samples were successfully printed using the process parameters summarised in Table 5. The samples are shown in Figure 12. The sample surfaces are rough, especially the top surface, where the melting traces were detected. The uneven surfaces were mainly due to the large powder size (d_50_ = 95 µm) used during the experiment. Using smaller powder size distribution can eliminate these problems.

**Figure 13:**
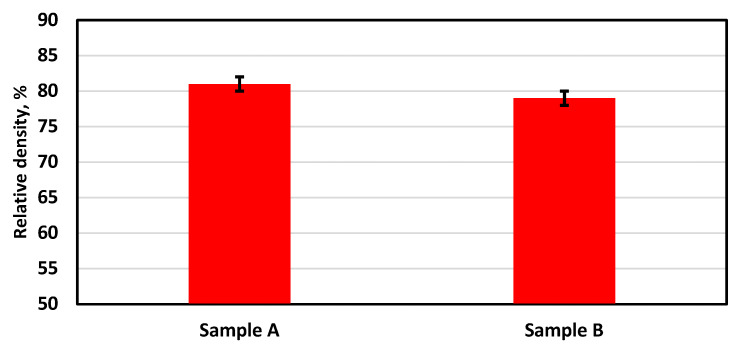
Relative density of alumina samples printed using the developed preheating system: with 20 W preheating laser power (Sample A) and 30 W (Sample B).

The Archimedes method was used to evaluate the relative density of the samples. Three measurements were carried out for each sample, and the average value was considered. Figure 13 shows the relative density value for samples A and B. Sample A exhibited a high relative density reaching more than 80%. This is considered the highest relative density achieved for alumina ceramic processed by PBF using a one-step AM process. This proves the effectiveness of the developed preheating system for PBF for ceramic materials. This finding will open the door to a breakthrough in AM of ceramics directly from pure powder without post-processing to reach the final product in one step. Sample B achieved a low relative density of 79%, and this may be due to the high preheating laser power used with this sample (the maximum preheating power of 30 W was used for this sample).

**Figure 14:**
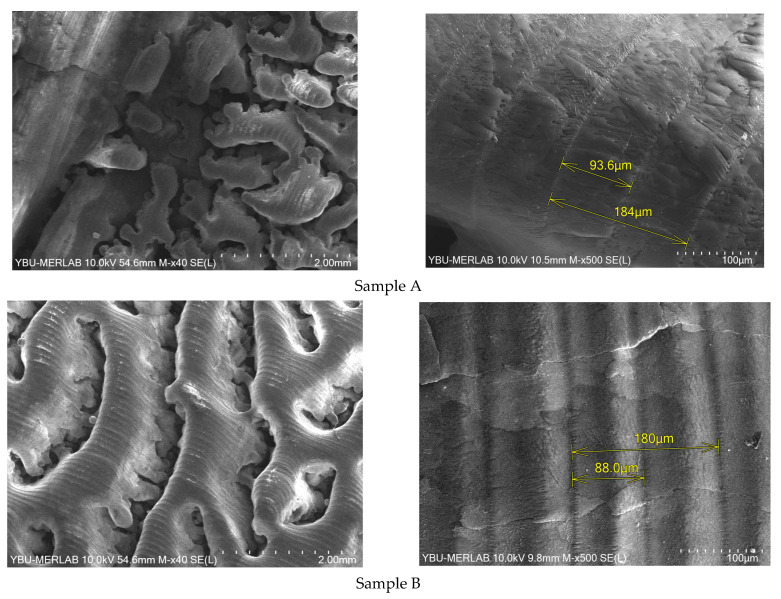
SEM images of alumina samples printed using the developed preheating system: with 20 W preheating laser power (Sample A) and 30 W (Sample B).

Figure 14 shows the SEM images for the top surface of alumina samples (without polishing). The samples experienced a level of porosity which may result from the large powder size distribution used as a feedstock. No cracks were detected on sample A, and the traces were well connected. The absence of cracks was mainly due to the low-temperature gradient developed on the powder bed due to preheating. Sample B experienced a transverse crack pattern and high porosity level. This is mainly due to the high preheating laser power used for the sample that resulted in its low relative density.

**Figure 15:**
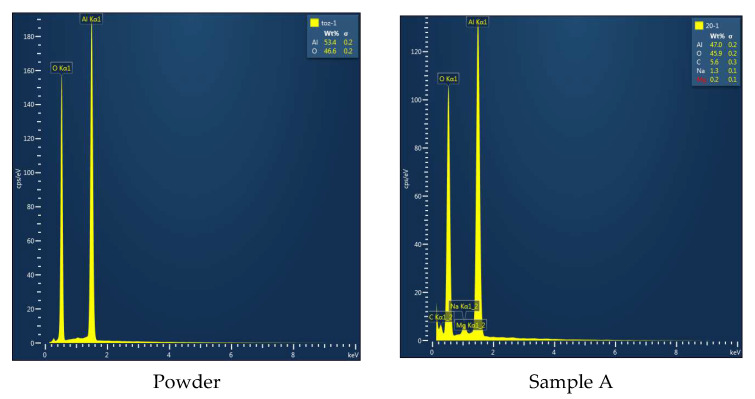
EDS of alumina powder and sample A printed using the developed preheating system.

**Figure 16:**
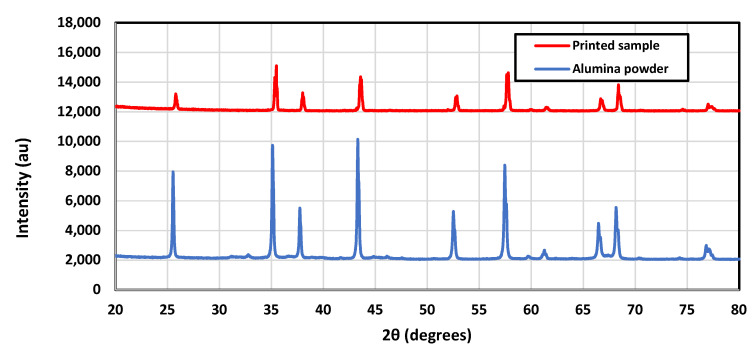
XRD spectra for the starting alumina feedstock and the printed sample (A).

Figure 15 shows the EDS of the alumina powder and sample A. Only aluminium and oxygen were shown in the EDS for alumina powder, while the EDS showed that other elements such as carbon and sodium were present in sample A. Since the samples were printed in the air, it is important to check how they might have been affected. The samples should have been printed in an inert gas environment to prevent contamination. This would require a printing environmental chamber which could be provided for future experiments. To accurately evaluate the effect of open-air printing, XRD analysis was performed for the starting feedstock (alumina powder). The printed sample (A) and the results are shown in Figure 16. It is clear that no change in the phases between the starting powder and the printed sample was observed, and no new phases were generated.

#### 3.3.2. Evaluation of the Developed Preheating System

**Figure 17:**
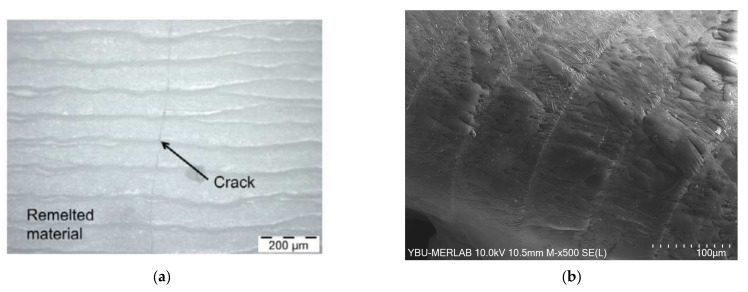
(**a**) SEM micrograph of the alumina–zirconia sample printed using the preheating system developed by Yves-Christian et al. [36]. (**b**) SEM micrograph of the alumina sample (A) printed using the preheating system developed in this study.

To show how effective the developed preheating system is, it was compared with previous preheating systems developed by Hagedorn et al. [36] and Liu et al. [37]. The preheating systems described in [36,37] were mainly based on preheating a large and fixed powder area. This technique has some disadvantages compared to our developed preheating system, which only preheats the printing path. The first disadvantage is that more energy is consumed in preheating a large area than in the printed area. Additionally, it cannot guarantee the proper and homogeneous preheating of the powder at the same rate; therefore, cracks were generated, as shown in Figure 17a. Using our developed preheating system, no cracks were detected, as shown in Figure 17b, due to the controlled and homogeneous preheating of the scanned printing path.

**Figure 18:**
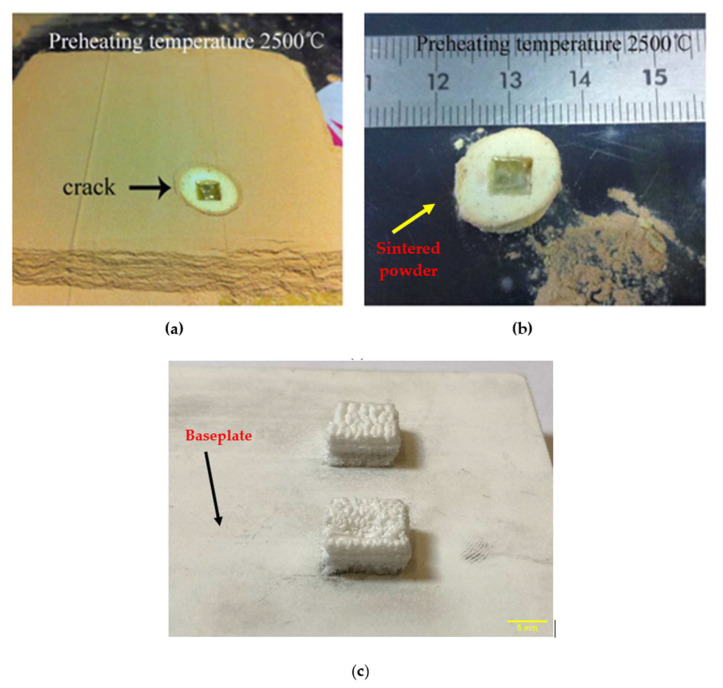
(**a**) In a powder bed YSZ sample printed using the preheating system developed by Liu et al. [37] (republished with permission). (**b**) Removed from powder bed, YSZ sample printed using the preheating system developed by Liu et al. [37] (republished with permission). (**c**) Sample printed using the preheating system developed in this study before removing from the baseplate.

Another disadvantage of the previously developed systems is that increasing the preheating temperature led to a sticky sintered powder surrounding the printed part, as shown in Figure 18a,b. This sticky powder can significantly affect the quality of the printed part and requires a post-processing step to be removed from the part surfaces. However, due to the nature of the developed preheating system, where only the current scanned path is preheated, no sticky powder can attach to the printed part, as shown in Figure 18c. This can prove the effectiveness of the developed preheating system in this study.

## 4. Conclusions

A novel preheating system for PBF of ceramic materials has been developed. The system included two laser systems: one for preheating and the other for melting/ sintering powder particles. The system’s uniqueness is that the two lasers simultaneously move along the same scanning path, but the preheating laser preceded the melting/sintering laser by a tiny time difference. This enabled the sintering/melting laser to scan a sufficiently preheated powder. In order to obtain appropriate process parameters, a numerical model was developed for this purpose. Alumina samples were successfully printed using the developed preheating system setup and the process parameters obtained from the numerical model. A relative density of more than 80% was achieved using a one-step AM process, considered the highest relative density of alumina obtained directly using the PBF technique. This finding provides a breakthrough in the AM of ceramics. A deep and detailed study of the process parameters (laser power, scanning speed, hatching distance, scanning strategies, and layer thickness) and mechanical performance evaluation will be considered for future work.

## Figures and Tables

**Table 1:** Physical and thermal properties of alumina powder.

Property	Value	Ref.
Density, kg/m^3^	3920	[41]
Specific heat J/kg·K	3 × 10^−13^ T^5^ − 3 × 10^−9^ T^4^ + 5×10^−6^ T^3^ − 0.0073T^2^ + 5.0097 T − 190.71, (T ≤ 2450) 1360, (T > 2450), (T, temperature in K)	
Thermal conductivity W/kg·K (T, temperature in K)	−3 × 10^−15^ T^5^ − 3 × 10^−11^ T^4^ − 10^−7^ T^3^ + 0.0002T^2^ − 0.203 T + 79.673, (T ≤ 2450) 5.5, (T > 2450), (T, temperature in K)	
Melting point, K	2327	
Latent heat of melting, J/kg	1,137,900	
Emissivity	0.7	
Stefan–Boltzmann constant, W/m^2^ K^4^	5.6704 × 10^−8^	
Thermal convection coefficient, W/m^2^ K^4^	200	
Absorptivity/CO_2_ laser	0.97	[42]
Absorptivity/Fibre laser	0.03	

**Table 2:** Model dimensions.

Parameter	Baseplate	Powder Layer
Length (mm)	4.4	4
Width (mm)	1.4	1
Thickness (mm)	0.5	0.06

**Table 3:** Laser sources specifications.

Item	CO_2_ Laser	Fibre Laser
Laser model	Universal URL-50	IPG-150 W
Spot size	170–180 µm	120–130 µm
Lens facial distance	160 mm	330 mm
Wavelength	10.64 µm	1.064 µm
Power	20 W, 30 W	150 W

**Table 4:** Top and cross-section melting contours.

Preheating Laser, W	Melting Laser, W	Temperature Contour	Melting Contour
20	150	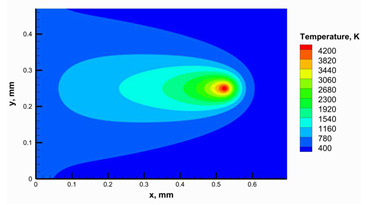	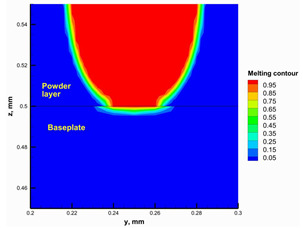
30	150	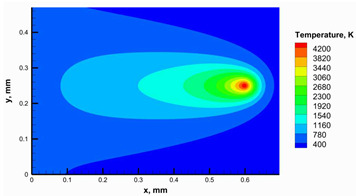	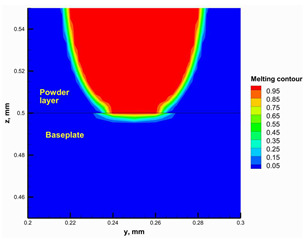

**Table 5:** The process parameters used to print different alumina samples.

Item	Sample A	Sample B
Preheating laser power, W	20	30
Melting laser power, W	150	150
Hatching distance, µm	50	50
Layer thickness, µm	60	60

## Data Availability

The authors confirm that the data supporting the findings of this study are available within the article.
